# The Effect of Accompanying In Situ Ductal Carcinoma on Accuracy of Measuring Malignant Breast Tumor Size Using B-Mode Ultrasonography and Real-Time Sonoelastography

**DOI:** 10.1155/2012/376032

**Published:** 2012-09-05

**Authors:** A. A. Soliman, S. Wojcinski, F. Degenhardt

**Affiliations:** ^1^Department of OB/GYN, Franziskus Hospital, 33615 Bielefeld, Germany; ^2^Department of OB/GYN, Shatby Maternity University Hospital, University of Alexandria, Alexandria 21526, Egypt

## Abstract

*Objectives*. Clinical estimation of malignant breast tumor size is critical for preoperative planning and is crucial for following up the tumor's response to the therapy in case she receives a neoadjuvant chemotharapy. Ductal carcinoma in situ (DCIS) accompanies about 25.4% of detected invasive breast cancers. The aim of this study was to examine the effect of the presence of DCIS on the accuracy of the ultrasonographic measuring malignant breast tumor size using B-mode and real time elastography. *Materials and Methods*. We recruited histologically confirmed breast cancer patients in a prospective observational study. *Results*. We recruited 50 breast cancer patients with a median age of 57.5 years. DCIS was confirmed to accompany 42% (*n* = 21) of the cases. Tumor size estimation using B-mode sonography (*P* < 0.001) as well as using real time elastography (*P* < 0.001). was statistically significant correlated to the actual tumor size. Presence of DCIS in 42% of our recruited patients affected the tumor size estimation using both methods thus losing the correlation between both estimations (*P* = 0.794). *Conclusion*. This study shows that the presence of DCIS significantly affects the accuracy of measuring the sizes of malignant breast tumors when using either B-mode ultrasonography or real time elastography.

## 1. Introduction

Breast cancer is the most common cancer affecting women in Germany. With 58 thousand new cases every year, it is estimated that every tenth woman in Germany will get breast cancer by the age of 74 [1, 2]. That is why breast cancer diagnostic technology occupies a very important position in the scientific community. Clinical estimation of malignant breast tumor size is very important. Most importantly, it is critical for preoperative planning, for whether a patient will receive a mastectomy, or be treated using a breast conservation operation. Without an accurate estimation of tumor size, the preoperative decision can be wrong [3]. Moreover, if tumor sizes are incorrectly estimated, the rate of reoperation will increase, with its risks, or a small tumor-free resection margin may be left behind that can lead to increased rates of local recurrence [4]. With the numbers of patients receiving neoadjuvant chemotherapy for treatment of breast cancer currently increasing, sonographic estimation of tumor size is very important for setting up the initial treatment plan, and then for following up the tumor's response to the therapy [5, 6]. Moreover, tumor size is one of the most important prognostic factors in breast cancer; accordingly, accurate tumor size estimation is considered a cornerstone in the treatment algorithm [7]. It is reported that ductal carcinoma in situ (DCIS) accompanies about 25.4% [8] of detected invasive breast cancers. That is why we considered it important to study the effect of the presence of DCIS, which is quite common, on the accuracy of measuring malignant breast tumor size, using both ultrasonographic diagnostic modalities.

## 2. Materials and Methods

This was a prospective observational study conducted in the Obstetrics and Gynecology Department, Franziskus Hospital, Bielefeld, Germany in the period between September and December 2011. The study was conducted on 50 confirmed unifocal breast cancer patients. The aim of the study was to study the accuracy of measuring the size of breast tumors using B-mode ultrasonography as well as real-time sonoelastography, in comparison to the real size of the tumor as confirmed by histological examination. The presence or absence of accompanying DCIS, and its effect on tumor size estimation using B-mode ultrasonography or real-time elastography were also studied. The ultrasound system platform used was a Hitachi EUB-8500 with an integrated real-time sonoelastography module (Hitachi Medical Systems, Wiesbaden, Germany). The ultrasound probe used was a linear probe, Hitachi EUP L54M, which is 50 mm wide with a maximum frequency of 13 MHz. Inclusion criteria: any patient presenting with breast cancer histologically confirmed through a tissue biopsy during the aforementioned time period was recruited. Each recruited patient received the routine B-mode ultrasonographic examination with recording of the maximum measurable tumor dimension. Real-time sonoelastography measurement of the tumor size, with recording of the maximal measurable tumor dimension, was performed as well. The measurements were taken just before performing the core tissue biopsy, and if the biopsy confirmed the diagnosis of breast cancer, the patient was recruited into our cohort, and the postoperative histopathology report was examined to obtain the required data, namely, the maximum dimension of the tumor and the presence or absence of accompanying DCIS in the specimen. The data was tabulated and analyzed using SPSS 13.0 for Windows. 

## 3. Results

We recruited 50 confirmed breast cancer patients during the aforementioned time period. The age of the recruited patients ranged from 30 to 87 years, with a median of 57.5 years. Invasive ductal carcinoma was present in 86% (*n* = 43) of the cases; invasive lobular carcinoma was present in only 12% (*n* = 6), while metaplastic carcinoma was present in just 2% (*n* = 1). The most common tumor grade was G2 76% (*n* = 38); G3 was present in 16% (*n* = 8), while the well-differentiated G1 was only present in 8% (*n* = 4). DCIS was confirmed to accompany 42% (*n* = 21) of the cases.

## 4. Discussion

**Figure 1:**
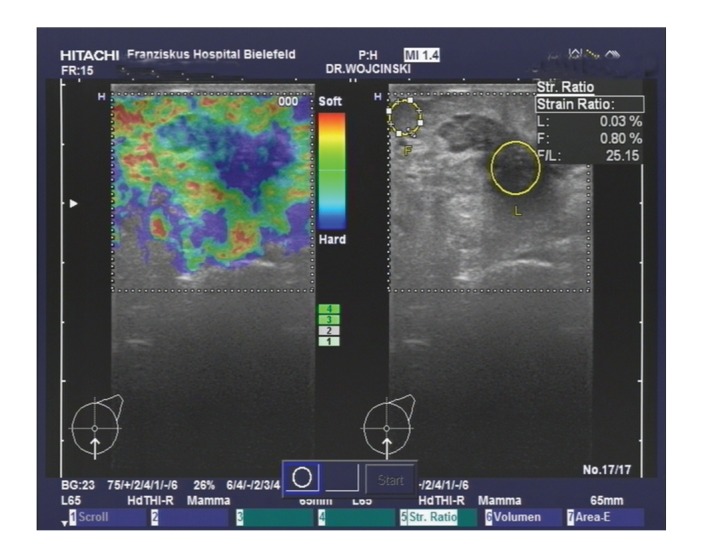
Sonoelastographic examination of histologically confirmed breast cancer in comparison to the morphologic features of B-mode ultrasonography.

**Figure 2:**
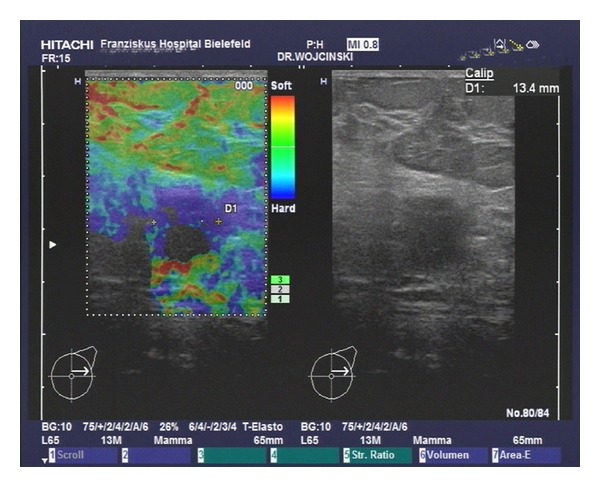
Measurement of the tumor size using sonoelastography.

Ultrasound imaging has been a gold standard imaging method for breast cancer tumor size estimation. Many published series have claimed that measurements of tumor size using conventional B-mode ultrasound imaging lead to underestimates of tumor sizes [9–11]; with increasing technical standards in the field of breast sonography, many recent reports have indicated accuracy in tumor size estimation is increasing [12–14]. Tumor size estimation using B-mode ultrasonography has been thoroughly studied and discussed in the literature. With every advance in the available technical standards, more and more publications and studies appear to demonstrate that B-mode ultrasonography is one of the most accurate methods for estimating breast cancer tumor size [15]. The accuracy of B-mode ultrasonography in detecting malignant breast lesion has been compared with the accuracy of other imaging modalities [10, 11, 15]; a near 100% sensitivity of B-mode ultrasonography in detecting palpable breast lesions is often reported. Moreover, the accuracy of determining tumor size using B-mode ultrasonography in comparison to the real tumor size as obtained through pathological examination has also been reported and proved valid [16–18]. With advances in ultrasound examination technology, and the advent and introduction of real-time elastography, breast cancer was one of the first major applications of this technology. Using real-time elastography to detect malignant breast lesions improved the diagnostic accuracy of conventional B-mode ultrasonography [19, 20]. A recent multicenter study proved that adding elastographic features to the breast mass feature analysis improved the specificity of breast ultrasound mass assessment without loss of sensitivity. This study showed an improvement in specificity from 61.1% to 77.4% when visual color stiffness was added to the ultrasonographic features usually examined and an improvement in specificity from 69.4% when the oval shape of the breast lesion and quantitative maximum elasticity were added to the features examined [18].  Figure 1 shows a sonoelastographic examination of one of our cohort's patients, and Figure 2 shows the measurement of the tumor size using sonoelastography. Most of the aforementioned reports however, focused on the sensitivity and specificity of B-mode ultrasonography as well as real-time elastography for detecting suspicious breast lesions and did not tackle the accuracy of measuring the sizes of the breast lesions detected. Meier-Meitinger and coworkers, in their work comparing breast cancer mass size assessments using ultrasonography, computerized tomography, mammography, and real-time elastography, demonstrated that the best and most accurate estimation of breast tumor size was provided using B-mode ultrasonography in cases of invasive ductal carcinoma. On the other hand, real-time elastography proved to possess superior accuracy in detecting breast mass size in instances of invasive lobular carcinoma. Both methods, however, proved superior to all other modalities in providing accurate measures of breast mass sizes when referenced against the final pathology report [15]. Our results (as illustrated in Table 1) conform with the findings of this study, where tumor size estimation using B-mode ultrasonography or real-time elastography was both significantly correlated to the real-tumor size as measured pathologically, reflecting accurate tumor size estimation. The difference between the pathologically confirmed real-tumor sizes and the measured values using B-mode ultrasonography, on one side, and real-time elastography, on the other, was also statistically correlated, reflecting the accepted accuracy of tumor size estimation by real-time elastography, as shown in Table 2. One of the drawbacks of our study is that the number of recruited patients was small, which did not give enough statistical power to the observations beforehand. The presence of DCIS accompanying breast cancer was confirmed in 42% of our recruited cohort. This value was above the published average of DCIS accompanying breast cancer, which is reported to be around 25.4% [8]. The presence of DCIS, however, had a significant impact on the accuracy of tumor size measurement. As shown in Table 3, in the presence of accompanying DCIS, no significant correlation could be found between the real tumor sizes as confirmed pathologically, and tumor size measurements using either B-mode ultrasonography or real-time elastography. This observation means that the presence of DCIS significantly affects the accuracy of measuring the sizes of malignant breast tumors, whether using B-mode ultrasonography or real-time elastography. DCIS is a complex pathologic entity [21] that could be better detected using mammography than ultrasonography [22], and our finding could be explained by the lack of notable morphologic changes in the case of accompanying DCIS to be detected sonographically.

## 5. Conclusions

This study shows that real-time elastography can be a reliable method for measuring the size of breast cancer tumors. However, the presence of DCIS significantly affects the accuracy of measuring the sizes of malignant breast tumors when using either B-mode ultrasonography or real-time elastography. More studies with larger numbers of recruited patients need to be designed in order to provide stronger statistical evidence for the above-mentioned observations, with greater statistical power.

## Figures and Tables

**Table 1:** It shows correlations between tumor sizes as measured using either B mode ultrasonography, or real-time sonoelastography, against the actual tumor sizes measured histologically.

			Tumor size by B mode	Tumor size on histology
Total	Tumor size by elastography	Rho	0.789	0.671
		*P*	<0.001*	<0.001*
	Tumor size by B mode	Rho		0.722
		*P*		<0.001*

rho (*ρ*): Spearman coefficient.

*Statistically significant at *P* ≤ 0.05.

**Table 2:** It correlates the differences in measurement in mm between the actual tumor sizes as measured in histopathology findings, and the sizes of the same tumors as estimated by means of either B-mode ultrasonography or real-time sonoelastography.

	Histology elastography	Histology-B mode	*P*
Range	−4.0–24.30	−8.30–22.0	0.004*
Mean ± SD	4.78 ± 6.70	3.25 ± 6.33	
Median	3.05	1.65	

*P*:*P* value for Wilcoxon signed ranks test.

Significant at *P *≤ 0.05.

**Table 3:** It shows the accuracy of sonographic estimation of the tumor size either by B-mode ultrasonography or by sonoelastography, in the presence and absence of accompanying DCIS, in relation to the actual tumor size as measured histologically through correlating the differences between the actual tumor size (histology report) and the size estimations by means of either B-mode or sonoelastography. Absence or presence of DCIS was confirmed through the histopathologic examination.

Presence of DCIS according to the histology report		Difference between maximum tumor size as measured by sonoelastography subtracted from maximum tumor size as measured histologically	Difference between maximum tumor size as measured by B-mode ultrasonography subtracted from maximum tumor size as measured histologically	*P*
	*N*	29	29	
	Range of the difference in tumor size estimation	−3.70–22.20	−5.0–22.0	0.001∗
No	Mean of the difference in tumor size estimation ± SD	6.06 ± 6.61	3.57 ± 6.66	
	Median	4.60	1.60	

	*N*	21	21	
	Range of the difference in tumor size estimation	−4.0–24.30	−8.30–16.50	0.794
Yes	Mean of the difference in tumor size estimation ± SD	3.02 ± 6.58	2.82 ± 5.96	
	Median	1.40	2.0	

*P*: *P* value for Wilcoxon signed ranks test.

*Significant at *P* ≤ 0.05.
